# Growth hormone receptor expression in human primary gastric adenocarcinoma

**DOI:** 10.7555/JBR.26.20110133

**Published:** 2012-09-15

**Authors:** Xiaodong Yang, Ping Huang, Feng Wang, Zekuan Xu, Xiaonin Wang

**Affiliations:** a Department of General Surgery, the First Affiliated Hospital, the Nanjing Medical University, Nanjing, Jiangsu 210029, China;; b Department of General Surgery, Jiangsu Hospital of Integrated Traditional Chinese and Western Medicine, Nanjing, Jiangsu 210028, China.

**Keywords:** growth hormone receptor, primary gastric cancer, proliferative index, apoptosis index

## Abstract

The aim of this study was to determine the expression of growth hormone receptor (GHR) in patients with primary gastric adenocarcinoma. We investigated 48 specimens of primary gastric adenocarcinoma and their corresponding normal gastric mucosa. Immunohistochemistry and reverse transcription-polymerase chain reaction (RT-PCR) were used to detect the expression of GHR. Immunohistochemical analyses revealed that GHR was expressed in human primary gastric adenocarcinoma (36/48, 75.0%) and appeared to be upregulated, compared to the normal mucosa (28/48, 58.3%, *P* < 0.001). A significant correlation was found between GHR expression and tumor stage (*P* < 0.001) and tumor differentiation (*P* < 0.001). The average positive rate of ki-67 in GHR-positive tumors was 16.06%, while the positive rate in GHR-negative tumors was 6.17% (*P* < 0.01). The average apoptosis index (AI) of GHR-positive tumors was 3.36%, which was significantly lower than that (7.33%) of GHR-negative tumors. In addition, 27 of 48 cases of tumors had *GHR* mRNA expression, while only 17 of all 48 cases of normal mucosa did so. Our results indicate that the frequency of GHR was significantly higher in primary gastric adenocarcinoma than that in normal gastric mucosa. GHR expression was significantly correlated with tumor differentiation and tumor grade. This finding supported a possible role of growth hormone in primary gastric adenocarcinoma pathophysiology.

## INTRODUCTION

Primary gastric cancer is a common malignancy in China. The overall mortality rate of gastric cancer in China has presented an increasing trend over the past two decades[1]. Diseases with a high level of growth hormone (GH), such as acromegaly, are associated with an increased risk of malignancies, including gastric cancer[2], suggesting that GH might be involved in the development of human primary gastric cancer. It has been previously demonstrated that growth hormone receptor (GHR) was expressed in human colorectal cancer and some other cancers[3]. However, GHR expression in primary gastric cancer has been rarely reported. In this study, we studied 48 cases of human primary gastric cancer and their corresponding normal mucosa using immunohistochemistry and reverse transcription-polymerase chain reaction (RT-PCR).

## SUBJECTS AND METHODS

### Patients and specimens

The study included 48 specimens of gastric cancer from patients who had surgery in our Hospital (Nanjing, China) from August 2003 to December 2003. The age and gender of patients, size, location, differentiation, and grade of tumors were recorded in all cases. Tumors and normal mucosa which was more than 10 cm distant from tumors were obtained in all cases. Hematoxylin-eosin (H&E) staining and immunohistochemical analysis were performed in all 48 cases. Serum samples were obtained from all 48 cases for the measurement of the plasma level of GH and IGF-1. For formalin-fixed paraffin-embedded tissue specimens, consecutive 4-µm sections were cut, and representative sections were stained for immunohistochemistry, H&E and terminal deoxynucleotidyl transferase (TdT)-mediated dUTP-biotin nick end-labeling (TUNEL) for apoptotic index (AI). Using the H&E-stained sections, the tumor histology and the grade of tumor differentiation were assessed according to the criteria of the World Health Organization (WHO)[5]. Tumors were staged according to the TNM staging system.

### Immunohistochemistry

For formalin-fixed and paraffin-embedded tissue specimens, consecutive 4-µm thick sections were cut and used for immunohistochemistry. The sections were immunohistochemically stained by the labeled streptavidin-biotin peroxidase method (LSAB2 Kit; Dako, Japan). The following primary antibodies were used: anti-GHR mouse monoclonal antibody (Novocastra Laboratories Ltd, UK), and anti-Ki-67 monoclonal antibody (Maxim, Shanghai, China). The sections were immersed for 10 min in 0.3% hydrogen peroxide/methanol to deplete endogenous peroxidase. Then, nonspecific binding sites were blocked with 0.3% of normal goat serum for 10 min. The primary antibody was then incubated with the sections overnight at 4°C. After washing with phosphate buffered saline (PBS, 0.01 mol/L, pH 7.4), biotinylated goat anti-mouse IgG was incubated with the tissue sections for 10 min at room temperature. After washing with PBS, the sections were sequentially incubated with a streptavidin peroxidase reagent for 10 min at room temperature. Finally, the reaction product was visualized by incubating the slides in a solution of 0.3% of hydrogen peroxide and 3-amino-9-ethylcarbazole (AEC) chromogen. The sections were counterstained with H&E. Negative controls included parallel sections treated without the primary antibody and adjacent sections from the same block but without the primary antibody which was replaced by PBS. The section of normal pancreas tissue treated under the same conditions was used as a positive control. To quantify GHR expression in sections, a semi-quantitative scoring system was used. The average percentage of positive-staining tumor cells was determined in at least five areas at ×400 magnification and assigned to one of the four categories: (a) 0≤5%; (b) 1=5%-33.3%; (c) 2=33.3%-66.6%; (d) 3=66.6%-100%. The immunoreactions were graded by at least two pathologists who were completely blind in this study.

### TUNEL staining

**Fig. 1:**
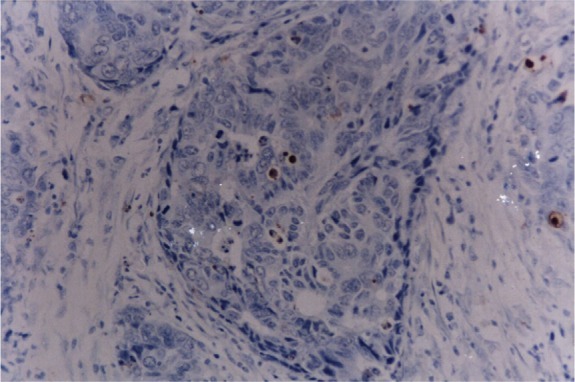
TUNEL staining of gastric cancer tissue (×400). The apoptotic cells were stained brown by TUNEL.

For detection of apoptotic cells, the AI was examined by the TUNEL method with an in situ cell death detection Kit POD (ISCDD, Boehringer Mannheim, Germany). The procedures were performed according to the manufacturer's protocol. Briefly, sections were deparaffinized, rehydrated, and washed with distilled water. Then, the sections were digested with 20 g/mL proteinase K (Boehringer Mannheim, Germany) for 15 min at room temperature. Endogenous peroxidase activity was blocked by incubating sections in 0.3% of hydrogen peroxide/methanol in PBS for 30 min at 37°C. Thereafter, the sections were incubated with terminal deoxynucleotidyl transferase for 60 min at 37°C to add the dioxigenin-conjugatd dUTP to the 3′-OH ends of fragmented DNA. Anti-digoxigenin antibody conjugated with peroxidase was added to the sections to detect the labeled nucleotides. The sections were stained with diaminobenzidine (DAB) and counterstained with H&E. The positive cells were identified, counted, and analyzed under a light microscope (***Fig. 1***). An average number of positive tumor cells per 100 tumor cells was scored at ×400 magnification, and at least 1000 tumor cells were included.

### RNA extraction

Forty-eight fresh frozen tissue samples of gastric cancer and normal gastric mucosa (80 mg per sample) were collected and minced. Total RNA was extracted from the fresh frozen tissue samples with the guanidinium thiocyanate method. RNA integrity was checked by agarose gel electrophoresis and monitoring A260/280 absorbance ratios before RT-PCR. RNA concentration was determined by spectrophotometric analysis at 260 nm before RT-PCR.

### RT-PCR

The RT-PCR method was described as follows. Briefly, one step RNA PCR Kit (Takara Bio Inc., Dalian, China) was used. The total reaction system (50 µL) included 5 mmol/L MgCl_2_, 0.8 U/µL RNase inhibitor, 0.1 U/µL AMV Reverse Transcriptase XL, 0.4 µmol/L of sense and antisense primers, 0.2 mmol/L dNTP, 0.1 U AMV-Optimized *Taq* polymerase and 5 µL×10 reaction buffer (TaKaRa Bio Inc., Dalian, China). One mg of RNA was converted into cDNA by incubating at 50°C for 30 min. The mixture was incubated at 94°C for 2 min to inactivate the Reverse Transcriptase XL. PCR amplification was performed for 30 cycles consisting of 30 sec at 94°C, 1 min at 60°C and 1 min at 72°C plus elongation 7 min at 72°C. After amplification, 20 µL of the PCR product were electrophoresed in a 2% (*W/V*) agarose gel. The PCR product had a size of 201 bp. For PCR, the following primers were used: 5′-GCTGCTGTTGACCTTGGC-3′ (sense) and 5′-ACCTCATCTGTCCAGTGG-3′ (antisense). PCR primers were designed according to the published GHR sequence[4]. β-actin was used as an internal control in the quantitative determination of GHR. The β-actin primers were designed as follows: sense primer: 5′-CATTTCCGGTGCACGATGGAG-3′ and the antisense primer: 5′-GCCATCCTAGCGTCTGGACCTG-3′.

### Measurements of the plasma level of GH and IGF-1

The plasma level of GH in human serum was determined by immunoradiometric assay (IRMA) (Daiichi Radioisotope, Tokyo, Japan). The plasma level of IGF-I was determined by Somatomedin C-RIA kit (Chiron, Yuka Medias Company Ltd., Tokyo, Japan).

### Statistical analysis

The Man-Whitney *U* test and Kruskal-Wallis test were used to examine the correlation between various clinical or pathologic parameters and GHR expression of tumors. The difference in GHR expression between tumors and normal gastric mucosa was determined using a Wilcoxon Signed Ranks Test. Student's non-paired *t* test was use to examine the difference of PI and AI between the tumors showing different GHR expression. A *P* value of less than 0.05 was considered statistically significant. All the calculations were performed by SPSS 10.0 for Windows (SPSS Inc., Chicago, IL, USA).

## RESULTS

### Patient demographic and disease characteristics

The characteristics of patients and tumors are summarized in ***Table 1***. The forty-eight patients ranged in age from 33 to 88 years (median, 62 years). There were 30 male and 18 female patients. Tumor size ranged from 4 to 150 cm^2^. Most tumors were poorly differentiated (22/48, 45.8%), and the remaining tumors were well (8/48, 16.7%) or moderately differentiated (18/48, 37.5%). This study included 9 cases of stage I, 9 cases of stage II, 24 cases of stage III, and 6 cases of stage IV.

### Immunohistochemistry

**Fig. 2:**
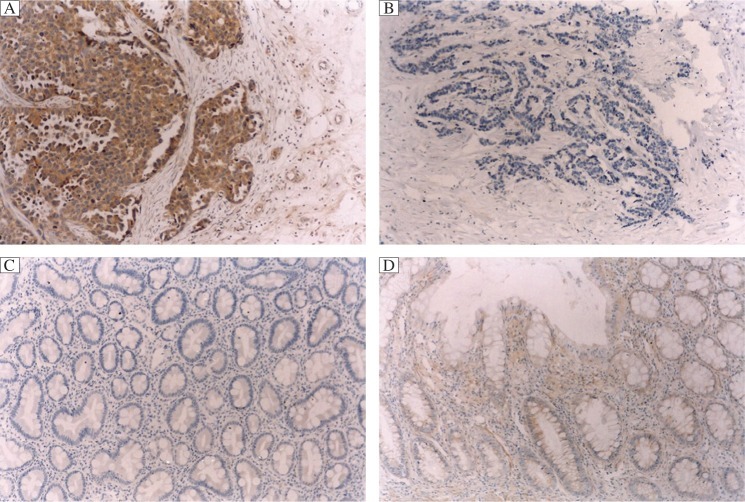
GHR staining of gastric cancer tissue and normal gastric mucosa. In contrast to tumors, which showed strong positive staining, normal gastric mucosa showed negatively or weakly positive staining, A: positive GHR staining of gastric cancers (×100); B: negative GHR staining of gastric cancers (×100); C: negative GHR staining of normal gastric mucosa (×100); D: positive GHR staining of normal gastric mucosa (×100)

The results of GHR immunostaining are summarized in ***Table 1***. Various degrees of cytoplasmic staining of tumor cells were seen in 36 of the 48 (36/48, 75%) analyzed tumors (***Fig. 2***), whereas the remaining 12 tumors (12/48, 25%) showed negative staining. In contrast to the tumors, the corresponding normal gastric mucosa showed negative or weakly positive staining (***Fig. 2***). A significant inverse correlation was found between GHR expression and the tumor stage (*P* < 0.001), and tumor differentiation (*P* < 0.001) (***Table 2***). Furthermore, there was no significant difference between GHR expression and age (*P* = 0.107), gender (*P* = 0.267), tumor location (*P* = 0.114), and tumor size (*P* = 0.098) (***Table 2***).

### Reverse transcription-polymerase chain reaction (RT-PCR)

Amplified fragments of an expected size of 201 bp were detected in 27 of all 48 tumor samples examined (27/48, 56.3%) (***Table 1***). *GHR* mRNA expression was also detected in 17 cases of normal gastric mucosa (17/48, 35.4%) (***Table 1***), whereas the remaining 31 cases had no *GHR* mRNA expression (***Table 1***).

**Table 1:** Clinical data, RT-PCR and immunohistochemical analysis of 48 cases of gastric tumors

Case	Age (year)	Gender	Tumor location	Tumor size(cm^2^)	Differentiation	Stage (I-IV)	GHR detection			
							RT-PCR		IHC(0-3)	
							T	N	T	N
1	68	F	M	2×2	well	II	+	+	2	2
2	68	M	U	4×5	poorly	IIIA	+	-	2	1
3	49	F	L	4×5	poorly	IIIB	+	+	3	2
4	52	M	U	3×4	moderately	IV	+	-	2	0
5	70	F	M	8×8	poorly	IIIA	-	+	2	2
6	68	M	L	2×3	poorly	II	-	-	1	0
7	79	F	U	4×5	poorly	IIIA	+	+	3	2
8	73	M	M	3×5	poorly	IIIB	+	+	3	1
9	54	M	M	4×4	well	IIIB	-	-	2	0
10	65	F	L	3×3	moderately	IB	-	-	0	0
11	53	M	M	4×6	poorly	IIIB	+	-	3	1
12	39	F	M	6×7	poorly	IIIB	+	+	3	3
13	33	F	U	4×3	moderately	IIIA	+	-	2	0
14	55	F	M	4×5	poorly	IIIB	+	+	2	2
15	34	F	L	2×4	well	IA	-	-	0	0
16	52	M	M	7×8	moderately	II	-	-	1	0
17	76	M	L	2×2	well	IA	-	-	0	1
18	50	F	L	3×4	moderately	II	-	-	0	0
19	63	M	U	4×5	poorly	IIIB	+	+	3	3
20	77	M	U	5×6	poorly	IV	+	+	3	2
21	53	M	M	12×10	moderately	IIIB	-	-	1	1
22	76	M	M	7×8	poorly	IIIA	+	+	2	3
23	79	M	M	2×2	poorly	IA	+	-	2	2
24	68	M	U	3×3	well	IA	-	-	0	0
25	69	M	L	8×5	moderately	IV	+	+	3	3
26	38	F	M	7×8	moderately	II	-	-	0	0
27	79	M	U	4×5	poorly	IIIA	+	-	2	1
28	68	F	L	3×3	moderately	IB	-	-	0	0
29	78	M	U	4×4	moderately	IV	+	+	3	2
30	68	M	M	8×5	poorly	IIIA	+	-	3	2
31	55	M	M	4×4	moderately	IV	+	+	2	2
32	70	M	L	15×10	poorly	IV	+	-	3	1
33	58	M	L	4×6	moderately	IIIA	-	-	0	0
34	47	M	M	10×12	poorly	IIIA	-	-	3	0
35	73	F	U	3×5	moderately	II	-	-	0	1
36	65	F	M	5×6	poorly	IIIA	+	-	2	0
37	48	M	U	4×3	moderately	IB	-	-	1	0
38	80	M	L	5×6	poorly	IIIA	+	+	3	3
39	46	F	M	5×7	moderately	IIIA	-	-	1	0
40	33	M	U	2×3	well	IA	-	-	0	0
41	44	M	M	7×7	poorly	IIIB	+	-	2	1
42	49	F	U	4×4	moderately	II	-	-	0	0
43	67	F	M	7×6	poorly	IIIB	+	+	3	3
44	76	M	M	3×5	moderately	IIIA	+	+	3	2
45	71	M	U	3×2	well	IB	-	-	0	0
46	63	M	U	5×4	moderately	II	+	-	2	1
47	74	M	L	4×5	well	II	-	-	1	0
48	80	F	M	08×11	poorly	IIIB	+	+	3	3

M: male; F: female; U: upper; M: middle; L: lower third of the stomach; Well: well differentiated; Moderately: moderately differentiated; Poorly: poorly differentiated; RT-PCR: reverse transcription-polymerase chain reaction; IHC: immunohistochemistry; T: gastric tumor; N: normal gastric mucosa.

### Correlation between mRNA expression and immunohistochemical staining

Immunohistochemical analysis revealed that the percentage of positive GHR expression in human gastric cancer was 75% (36/48), and RT-PCR revealed that the percentage of positive *GHR* expression was 56.25% (27/48) (***Table 1***). All 27 cases of tumors which expressed *GHR* mRNA showed positive staining in immunohistochemistry. Only 9 cases of tumors, which were graded 1+ to 2+ in immunohistochemistry, showed negative *GHR* mRNA. However, the remaining 12 cases were negative in both RT-PCR and immunohistochemistry.

### Correlation between PI, AI, GH, IGF-1 and GHR expression in primary gastric adenocarcinoma

The PI was 6.17%±1.85%, 10.50%±1.05%, 13.36%±2.41%, and 20.50%±4.08% in four groups of primary gastric adenocarcinoma graded 0, 1, 2, and 3 in GHR expression, respectively (***Table 3***). While the AI of the four groups was 7.33%±2.71%, 5.33%±1.86%, 4.50%±1.02%, and 1.63%±1.36%, respectively (***Table 3***). There were statistically significant differences between the four groups. The more intense the expression of GHR in primary gastric adenocarcinoma was, the higher the PI and the lower the AI were found.

There were no statistically significant differences in plasma levels of GH and IGF-1 between the different groups of GHR expression (***Table 4***).

## DISCUSSION

In addition to the effect on growth, GH shows a wide range of effects on the metabolism of carbohydrate[6], lipid[6],[7], and protein[8], and plays an important role in maintaining normal body composition. GH is widely used clinically and was approved by the U. S. Food and Drug Administration (FDA) in the therapy of GH deficiency in adults[9] and human immunodeficiency virus (HIV) wasting syndrome[10],[11]. The well-known anabolic actions of GH have also led to use in patients in severe catabolic states, such as surgery[12], trauma[13], burns[14], intestinal fistula[15] and short bowl syndrome[16],[17]. GH has been also used to overcome apparent resistance and reverse protein catabolism that leads to muscle weakness, impaired wound healing[18] and immune responses[19],[20], resulting in increased mortality. But data from Japan suggested a possibly increased risk of leukemia in children treated with GH. Physicians also worried that GH might increase the risk of other tumors or tumor recurrence for people who had developed tumors (such as gastric cancer) previously. It has been reported that GHR is expressed in colorectal[3] and liver cancers[21]. In this study, the results clearly demonstrated that GHR was expressed in human primary gastric cancer. Moreover, our immunohistochemical analysis demonstrated that GHR was expressed more highly in tumors than in normal mucosa. Thus, the adjacent normal mucosa showed no or weak immunoreactivity for GHR, and almost all cases of tumors showed strong or moderately diffuse cytoplasmic staining. The GHR immunoreactivity was predominantly cytoplasmic in our study, with occasional weak nuclear staining. This is interesting since nuclear translocation of GHR may be induced by GH simulation. GH, possibly via insulin-like growth factor (IGF)-1, exerts its effect during development of primary gastric cancer.

**Table 2:** Association between growth hormone receptor (GHR) expression and clinicopathological parameters

Clinicopathological parameters	Number of cases	GHR				*P*
		0	1	2	3	
Age (year)						
< 62	20	6	4	6	4	
≥62	28	6	2	8	12	0.107
Gender						
Male	30	5	5	9	11	
Female	18	7	1	5	5	0.267
Stage						
I	9	7	1	1	0	
II	9	4	3	2	0	
III	24	1	2	9	12	
IV	6	0	0	2	4	< 0.001*
Differentiation						
Well	8	5	1	2	0	
Moderately	18	7	4	4	3	
Poorly	22	0	1	8	13	< 0.001*
Size (cm^2^)						
≤30	34	11	3	11	9	
> 30	14	1	3	3	7	0.098
Location						
Upper	15	5	1	5	4	
Middle	21	1	3	9	8	
Lower	12	6	2	0	4	0.114

In this study, there was an association between GHR expression and tumor stage and tumor differentiation. The more intensely GHR was expressed in primary gastric adenocarcinoma, the higher the PI and the lower the AI was found. These findings suggest that increased GHR expression might be involved in the proliferative potential and aggressive biological behavior of primary gastric adenocarcinoma. Thus, recombinant human GH (rhGH) should be carefully used in patients with gastric cancer. In contrast, GH antagonists[22],[23] might be used in gastric cancer showing positive GHR expression in the future.

**Table 3:** Association between growth hormone receptor (GHR) expression and clinicopathological parameters by using multiple regression models

Clinicopathological parameters	GHR				*P*
	0	1	2	3	
Stage					
I	7	1	1	0	
II	4	3	2	0	
III	1	2	9	12	
IV	0	0	2	4	0.003*
Differentiation					
Well	5	1	2	0	
Moderately	7	4	4	3	
Poorly	0	1	8	13	0.017^#^
PI	6.17±1.85	10.50±1.05	13.36±2.41	20.50±4.08	0.030^#^
AI	7.33±2.71	05.33±1.86	04.50±1.02	01.63±1.36	0.034^#^

AI: apoptotic index; GHR: growth hormone receptor; PI: proliferation index. *P < 0.01, ^#^*P* < 0.05.

**Table 4:** Association between GHR expression and GH and IGF-1 in human gastric cancers

GHR expression	Number of cases	GH (ng/mL)	IGF-1 (µg/L)
0	12	0.67±1.06	369.8±189.1
1	6	0.70±1.21	388.3±205.2
2	14	0.66±1.31	357.2±190.4
3	16	0.74±1.08	373.5±179.9
		*P >* 0.05	*P >* 0.05

GHR: growth hormone receptor; GH: growth hormone; IGF-1: insulin-like growth factor-1.

Lin *et al*.[24] investigated the behavior of a tumor with various expression of GHR using gastric cancer cell lines. The immunohistochemistry results showed that GHR expression of SGC-7901 was strongly positive (GHR(+++)), while GHR expression of MKN-45 was negative [GHR(-)]. After rhGH treatment, tumor growth was significantly increased in SGC-7901 (GHR(+++)) tumor-bearing mice, However, in MKN-45 [GHR(-)] tumor-bearing mice, tumor growth was not significantly increased by rhGH. These findings suggest that the level of GHR expression is critical in influencing the effectiveness of rhGH on promoting the growth of gastric cancer and angiogenesis. It is reported that rhGH may promote the activation of tumor angiogenesis factors through the Jak-2-STAT3 signaling pathway.

GHR is widely distributed in the gastrointestinal tract[25], and GH takes effect on target tissues by binding to GHR[26]. In the stomach, GHR is mainly distributed among parietal and chief cells. Chronic atrophic gastritis (CAG) is a gastric precancerous lesion and listed as the first factor for the prevention of gastric cancer by the WHO. The levels of GH/GHR expression in patients with CAG were significantly lower than normal subjects. Decreased levels of GH and GHR had an adverse effect on the progression of CAG[27].

Despite that significant assoication between GHR expression levels was detected by immunohistochemistry and RT-PCR, a significant assoication between GHR expression and tumor grade, and tumor differentiation was found only with immunohistochemistry. A semi-quantitative RT-PCR might overcome this discrepancy. This study indicates that GH and GHR play a role in human primary gastric cancer, but the exact mechanism involved remains unclear. We speculated that the autocrine/paracrine mechanisms might be involved.

In conclusion, our study, which provided evidence of GHR expression and upregulation in human gastric cancer, indicated a role for GHR signaling in human gastric cancer. However, GHR function was not examined in this study. Furthermore, the in vivo and in vitro proliferative effect of GH to gastric cancer and its underlying mechanisms were also not investigated. To understand the underlying mechanisms, further studies are necessary, including the analysis of benign gastric lesions and precancerous conditions.
